# Combining Hydrology and Mosquito Population Models to Identify the Drivers of Rift Valley Fever Emergence in Semi-Arid Regions of West Africa

**DOI:** 10.1371/journal.pntd.0001795

**Published:** 2012-08-21

**Authors:** Valérie Soti, Annelise Tran, Pascal Degenne, Véronique Chevalier, Danny Lo Seen, Yaya Thiongane, Mawlouth Diallo, Jean-François Guégan, Didier Fontenille

**Affiliations:** 1 CIRAD, UPR AGIRs, Montpellier, France; 2 CIRAD, UMR TETIS, Montpellier, France; 3 CIRAD, UR SCA, Montpellier, France; 4 ISRA/LNERV, BP 2057, Dakar-Hann, Senegal; 5 Institut Pasteur de Dakar, BP 220, Dakar, Senegal; 6 IRD, UMR MIVEGEC (IRD 224, CNRS 5290), Université de Montpellier, Montpellier, France; 7 EHESP, Montpellier, France; NASA Goddard Space Flight Center, United States of America

## Abstract

**Background:**

Rift Valley fever (RVF) is a vector-borne viral zoonosis of increasing global importance. RVF virus (RVFV) is transmitted either through exposure to infected animals or through bites from different species of infected mosquitoes, mainly of *Aedes* and *Culex* genera. These mosquitoes are very sensitive to environmental conditions, which may determine their presence, biology, and abundance. In East Africa, RVF outbreaks are known to be closely associated with heavy rainfall events, unlike in the semi-arid regions of West Africa where the drivers of RVF emergence remain poorly understood. The assumed importance of temporary ponds and rainfall temporal distribution therefore needs to be investigated.

**Methodology/Principal Findings:**

A hydrological model is combined with a mosquito population model to predict the abundance of the two main mosquito species (*Aedes vexans* and *Culex poicilipes*) involved in RVFV transmission in Senegal. The study area is an agropastoral zone located in the Ferlo Valley, characterized by a dense network of temporary water ponds which constitute mosquito breeding sites.

The hydrological model uses daily rainfall as input to simulate variations of pond surface areas. The mosquito population model is mechanistic, considers both aquatic and adult stages and is driven by pond dynamics. Once validated using hydrological and entomological field data, the model was used to simulate the abundance dynamics of the two mosquito species over a 43-year period (1961–2003). We analysed the predicted dynamics of mosquito populations with regards to the years of main outbreaks. The results showed that the main RVF outbreaks occurred during years with simultaneous high abundances of both species.

**Conclusion/Significance:**

Our study provides for the first time a mechanistic insight on RVFV transmission in West Africa. It highlights the complementary roles of *Aedes vexans* and *Culex poicilipes* mosquitoes in virus transmission, and recommends the identification of rainfall patterns favourable for RVFV amplification.

## Author Summary

Rift Valley fever (RVF) is a zoonotic disease that affects domestic livestock and humans. During inter-epizootic periods, the main infection mechanism is suspected to be through bites by infected mosquitoes, mainly of *Aedes* and *Culex* genera. In East Africa, RVF outbreaks are known to be closely associated with heavy rainfall events, unlike in the semi-arid regions of West Africa where the drivers of RVF emergence remain poorly understood. This study brings mechanistic insight to explain why reported RVF outbreaks in Northern Senegal cannot be correlated directly to rainfall. This is done through the use of a rainfall-driven model of RVF vector populations that combines a hydrological model to simulate daily water variations of mosquito breeding sites, with mosquito population models capable of reproducing the major trends in population dynamics of the two main vectors of RVF virus in Senegal, *Ae. vexans* and *Cx. poicilipes*. Results show that RVF occurs during years when both species are present simultaneously in high densities. Simulations of inter-annual variations in mosquito populations successfully explained the dates of RVF outbreaks observed between 1961 and 2003.

## Introduction

Rift Valley fever (RVF) is a vector-borne disease caused by a virus (RVFV) belonging to the *Bunyaviridae* family, genus *Phlebovirus*, that affects domestic livestock (*e.g.*, sheep, cattle, camels, and goats) and humans. In humans, RVF can take different forms [1]. Most human cases are characterized by a ‘dengue-like’ illness with moderate fever, joint pain, and headache. In its most severe form, the illness can progress to hemorrhagic fever, encephalitis, or ocular disease with significant death rate. In livestock, it causes abortion and high mortality of newborns and thus induces important direct and indirect economic impacts.

Since the first isolation of RVFV in Kenya in 1930 [2], major RVF outbreaks have been reported in Egypt in 1977–1978 [3] and 1993 [4], in the Senegal River Valley in 1987 [5], [6], in Madagascar in 1990 [7] and 1992 [8], and in northern Kenya and Somalia in 1997, 1998 and 2007 [9]. In 2000, RVF cases were reported for the first time outside the African continent, in Saudi Arabia and Yemen [10]. Recently, a new wave of RVF epidemics occurred in 2006 and 2007 in East Africa (Kenya, Somalia and Tanzania) [11], [12], in Sudan in 2007 [13], in Madagascar in 2008 [14], and in Southern Africa in 2010 [15].

Two main modes of transmission of RVFV are suspected: *i)* a direct transmission from infected ruminants to healthy ruminants or humans, *(ii)* an indirect transmission through the bites of infected mosquito vectors [16]. The respective contribution of these different transmission routes remain unevaluated [17]. However, it is assumed that the transmission by the bite of infected mosquitoes is the main infection mechanism during inter-epizootic periods [18].

The number of mosquito species potentially involved in RVFV transmission is very large (more than 30 species), with the main vectors belonging to the *Aedes* and *Culex* genera [19]. Because mosquitoes are highly dependent on environmental conditions, the distribution in space and time of RVF is also related to climatic and landscape features. Until now, the ecological areas associated with RVFV transmission were either irrigated or flooded areas located in bushed or wooded savannas of semi-arid areas [20], although a recent study on RVF outbreaks in Madagascar showed possible transmissions in a temperate and mountainous region [17]. In semi arid areas, natural water bodies which are generally full during the rainy season allow the development of *Aedes* and *Culex* species [20], [21]. Based on this, climate based models have been developed to predict RVF outbreaks in Eastern Africa [22], [23], and a strong correlation was found between extreme rainfall events and RVF outbreak occurrences in the Horn of Africa [24].

**Figure 1:**
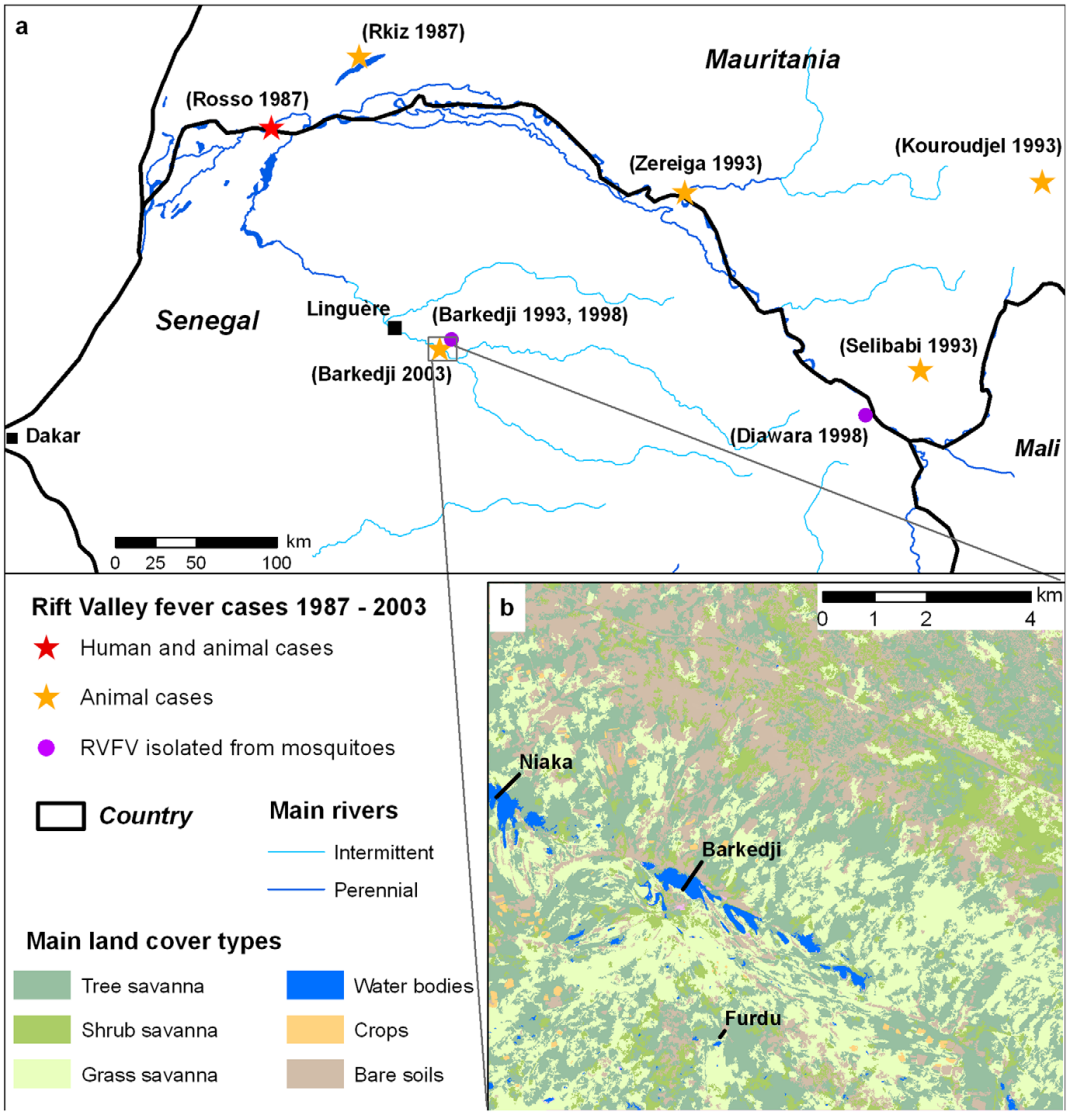
Study area. a) Location of Rift Valley fever outbreaks reported in Senegal [21], [27], [28], [29] and Mauritania [25], [26] (1987–2003). b) Land cover map showing location of ponds (in blue) and mosquito trap locations near Barkedji village, Ferlo Region, Senegal.

In West Africa, there is strong evidence that the disease is endemic [18]: different RVF outbreaks were reported in ruminants since the severe outbreak in the Senegal River basin in 1987 [25], [26], [27], [28], and RVFV was isolated from mosquitoes [21], [29] (Figure 1a). However, using a statistical approach, the correlation found in East Africa is not valid in the semi-arid regions of West Africa [30], [31] where the drivers of RVFV transmission dynamics remain poorly understood. There, temporary water bodies (ponds) constitute the main oviposition sites of different mosquito species [32], [33] and mosquito population dynamics are assumed to mainly depend on water availability and on pond dynamics, themselves driven by rainfall [34].

In this study, we use a mechanistic modelling approach to better understand the dynamics of RVF transmission in Northern Senegal, in relation to the population dynamics of its two main mosquito vectors in Senegal, *Aedes (Aedimorphus) vexans arabiensis* [21], [33] and *Culex poicilipes* [29]. These two species are considered as the main RVF vector in the area because *i)* they were proven experimentally to be competent for RVF virus transmission [35], [36], [37]; *ii)* they were frequently found infected in nature and are the most abundant species in our field site [21], [38]; *iii)* their interaction with the RVF vertebrate hosts (sheep, goats, and cattle) is very important [39]. The dynamics of the two vector species is modelled by combining a hydrological model of the dynamics of the water bodies, with mosquito population models describing different stages of the mosquito life cycle. Once calibrated and validated on recent rainfall, pond water levels, and entomological data, the combined model can be used to simulate the evolution of the two species' populations during the period 1961–2003, using only rainfall data as input. The comparison of model simulations with recorded prevalence rates and RVF outbreaks in the region is then analyzed and discussed.

## Methods

### Study area

The study area is an agropastoral zone of northern Senegal (Figure 1b). It is representative of the Ferlo region and is characterized by a complex and dense network of ponds that are filled during the rainy season (from July to mid-October). These water bodies are focal points where humans and livestock have access to water during the rainy season and are also the main breeding sites for *Aedes vexans arabiensis* and *Culex poicilipes* mosquitoes.

### Hydrologic model overview

We used a hydrologic pond model that simulates daily spatial and temporal variations (surface, volume, and height) of temporary ponds in arid areas [40]. The model consists in a daily water balance model taking into account the contribution from direct rainfall, the runoff volumes of inflows and the water loss through evaporation and infiltration. The relation between water volume, surface and height of a given pond depends on the 3D shape of that pond and is modelled by two volume-depth and area-depth empirical equations. Parameters of the model were estimated using detailed bathymetry of representative ponds of the study area and remotely sensed data such as a Digital Elevation Model (DEM) and a very high spatial resolution Quickbird image.

The model was calibrated and validated with field data (water height data and shape profile) collected during the rainy season 2001 and 2002 in the Barkedji area. The application of the model to the ponds (98) of the study area gave fair results both for water height and water area predictions. The comparison of simulated and observed water areas show significant correlations with a coefficient of determination (r^2^) of 0.89. More details of the hydrologic model are given in [40].

In this study, two sets of rainfall data were used as model input: *i)* daily rainfall data recorded during the rainy seasons (July–December) 2002 and 2003 with an automatic meteorological collector located in Barkedji village (Figure 1b); and *ii)* daily rainfall data recorded from January 1961 to December 2001 by the Linguère meteorological station located 30 km from Barkedji (Figure 1a). The output of interest of the hydrologic model for modelling mosquito population dynamics is 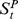, the water surface of any pond *P* at time *t*.

### Bioecology of *Aedes vexans* and *Culex poicilipes* mosquitoes

The mosquito life cycle involves aquatic (egg, larva, and pupa) and aerial (adult) stages. It begins with an egg, which hatches as a larva. Depending on the species and environmental conditions, hatching may occur immediately or may be delayed. The larvae then mature through four stages before entering pupation. After pupation, the mosquito emerges as an adult (imago) at the surface of water. Adults rapidly mate after emergence and females then seek a blood meal necessary for developing their eggs. Following egg development of about three days, females lay eggs on specific humid surfaces (oviposition sites), proceed to a new blood meal, and perform a new gonotrophic cycle, which corresponds to the period between 2 successive egg layings.

The bioecology of *Ae. vexans* and *Cx. poicilipes* differs. *Cx. poicilipes* eggs are deposited directly on water surfaces and immediately proceed through development into larvae; they do not survive dessication. In contrast, *Ae. vexans* females lay their eggs on the soil just above the current water level [33]. To hatch, the eggs must first dry out for a minimum number of days before being submerged in water. Moreover, in dry Sahelian regions, *Cx. poicilipes* populations may survive unfavourable conditions of the dry period as adults in dormancy (diapause) whereas *Ae. vexans* survive as eggs in desiccated mud, that will hatch during the next rainy season [33].

### The mosquito population model

In the context of data scarce regions, we developed a simple model that captured the main features of *Ae. vexans* and *Cx. poicilipes* dynamics at the scale of a pond. The sole dynamic input was the water surface area of pond *P* at a daily time step *t*, written as 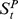. Only female mosquitoes are modelled and the two mosquito populations of each pond are assumed independent. We followed the theoretical framework proposed by Porphyre et al. [41] for *Cx. poicilipes* populations, and we extended this model to better take into account specificities of the bioecology of *Ae. vexans*.

The dynamics of the number of adult female mosquitoes of pond *P*, time step *t*, 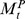, is described by:
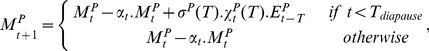
(1)where 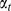 is the daily mortality rate, *T* the developmental period, *i.e.* the elapsed time during which a newly hatching egg undergoes its development until the emergence of an adult, 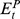 the number of hatching eggs in the pond *P*, time step *t*, and *T_diapause_* the date when mosquitoes enter into diapause. The production rate of new adults from a pool of hatching eggs is expressed as the product of the mosquito production capacity of the breeding site, 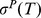, and of the availability function of the pond *P*, 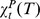.

#### Production rate

From a pool of hatching eggs at earlier time *t-T*, a proportion 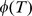 survives the maturation and transformation stages up to the time of emergence *t*, with 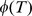 the pre-imago survival probability depending on the developmental period *T* and the daily larval survival rate 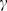:
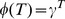
(2)Simultaneously to the maturation and transformation phases, the breeding site (pond *P*) undergoes changes from a surface 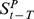 to 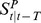, where 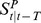 represents the smallest surface during the developmental period that still contains stages susceptible of leading to emergence of adults:
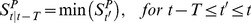
(3)Thus, at time *t*, only a fraction 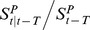 of surviving pupa 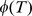 have a chance 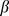 of giving rise to emergence of adults, out of which a proportion 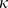 are females.

As a result, the production rate of new mosquitoes from a pool of hatching eggs is given by
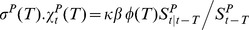
(4)With
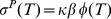
(5)and
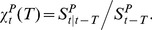
(6)


#### *Culex poicilipes* hatching eggs

Considering the very high rate of hatching eggs of *Culex* mosquitoes [42], the number of hatching eggs 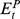 is calculated as the number of eggs laid by the female mosquitoes at time *t* on pond *P*.

Let 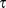 be the length of the gonotrophic cycle. At each time step *t*, only a fraction 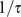 of the adult female mosquito population oviposits, with 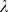
eggs laid per female. The success of oviposition at pond P is derived from the fraction 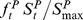 of the pond surface available for mosquito laying, 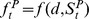 being a scaling factor to take into account that females only oviposit at a given inner distance *d* from the pond border. Considering 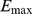 the maximum egg density, the number of *Cx. poicilipes* hatching eggs is calculated as:
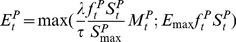
(7)


#### *Aedes vexans* hatching eggs

As for *Cx. poicilipes*, the number of eggs laid by *Ae. vexans* female mosquitoes in the humid surface surrounding the pond depends on the number of female mosquitoes *M*, the number of eggs laid by female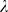, and the length of the gonotrophic cycle 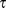. But the number of hatching eggs from a pool of eggs laid by *Aedes* female mosquitoes at time *t-k*, 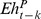, will be null if *k* is less than the minimum desiccation period *T_d_* or if the eggs were submerged in water before achieving the minimum desiccation period. Moreover, the eggs will only hatch at time *t* if 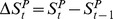, the pond surface variation between *t* and *t-1*, is positive. In that case, the potential hatching surface is 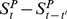, with *t'* defined such as 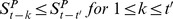 and the dynamics of the *Aedes* hatching eggs 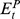 is described by:
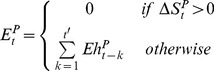
(8)with 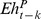, the number of hatching eggs from a pool of eggs laid by *Aedes* female mosquitoes at time *t-k*, being derived from the number of eggs laid using a normal distribution to describe the distribution of the eggs around the pond. 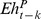 will be null if *k* is less than the minimal length of desiccation period (*k<Td*) or if there exists (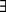) a time step *j*, comprised between *t-k* and *t-k+T_d_*, such as the water surface at time *j* (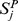) is greater than the water surface at time *t-k* (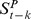) (in that case the eggs are submerged in water before achieving the minimum desiccation period):
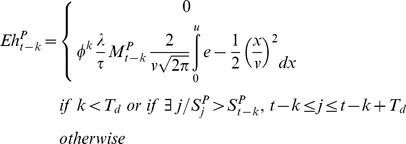
(9)with φ the daily survival rate of eggs in desiccation phase, 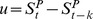 and 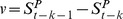. The possible multiple hatches of a single brood after successive floodings were here neglected, as the majority of *Ae. vexans* larvae usually emerged after the first flooding [43], [44].

#### *The daily mortality rate*

The daily mortality rate of adult mosquitoes was derived from the Davidson's method [45]:
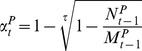
(10)where the number of nulliparous females 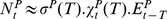, and τ the length of the gonotrophic cycle.

Parameters and variables of the model are summarized in Table 1.

**Table 1:** Variables and biological parameters of the mosquito population model.

	Parameters and variables	Value/Range of values/Equation*		Reference
*Input variable*				
S	Pond surface area (m^2^)	*74≤S≤347400*		[58]
*State variables*				
M	Number of adult female mosquitoes	*(Eq.1)*		
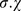	Production rate of new adults from a pool of hatching eggs	*(Eq.4)*		
E	Number of hatching eggs	*Cx. poicilipes*	*(Eq.7)*	
		*Ae. vexans*	*(Eq.8)*	
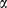	Mortality rate	*(Eq.10)*		
*Parameters*				
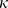	Sex ratio	*Cx. poicilipes*	0.5	[59]
		*Ae. vexans*	0.5 [0.42–0.53]	[60]
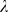	Number of eggs laid/female/day	*Cx. poicilipes*	[100–200] *	[59]
		*Ae. vexans*	100 [100–120]	[61]
*τ*	Gonotrophic cycle duration (days)	*Cx. poicilipes*	3 [3]–[4]	[33], [38]
		*Ae. vexans*	3 [3]–[4]	[33], [38]
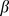	Transition probability from pupae to imago emergence	*Cx. poicilipes*	0.75	[59]
		*Ae. vexans*	0.60	[62]
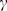	Daily larval survival rate	*Cx. poicilipes*	0.90 *	[59]
		*Ae. vexans*	0.80 *	[62]
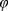	Daily survival rate of *Aedes* eggs in desiccation phase	*Ae. vexans*	[0.83–99.7] *	[63]
*T*_d_	Minimal length of desiccation period for *Aedes* eggs (days)	*Ae. vexans*	[5]–[7] *	[33]
*T*	Transformation time (days)	*Cx. poicilipes*	[9]–[17] *	[60], [64]
		*Ae. vexans*	[3]–[10] *	[21], [60]
*Emax*	Eggs maximum density/m^2^	*Cx. poicilipes*	[7 10^5^–1.5 10^6^] *	[59]
*d*	inner distance (m) from the pond border defining the laying area of *Culex* on the water surface	*Cx. poicilipes*	1	[65]

* : See calibration.

### Initial conditions and simulations

The hydrologic model and both *Cx. poicilipes* and *Ae. vexans* models were run for two ponds in the study area, Niaka and Furdu (Figure 1b). The two ponds were considered representative of the water bodies in the area, Niaka (363 525 m^2^) being a large pond located in the main stream of the Ferlo Valley, and Furdu (9 603 m^2^) being a smaller pond located outside the main stream [40].

The initial *Cx. poicilipes* adult population was defined proportionally to the pond perimeter covered by vegetation, with an initial density of adults of 1 adult.m^−1^. The initial number of *Ae. vexans* eggs was defined proportionally to the pond surface, with an initial density of 1000 eggs.m^−2^. Simulations started June 1^st^, at the beginning of the rainy season. The date of diapause was October 1^st^, according to [46].

### Sensitivity analysis

A sensitivity analysis was carried out to assess the robustness of the mosquito population model. We used the OAT (one-factor-at-a-time) Morris's method [47], as revised by Campolongo (1999), allowing the estimation of the two-factor interaction [48], [49]. The input parameters and their ranges based on the literature data were used in the analysis. When information was unavailable, the parameters space variation was defined using nominal values ±10% and a uniform distribution. Three outputs have been tested for each species: *(1)* the cumulated annual abundance, *(2)* the maximum abundance, and *(3)* the date of the peak of abundance.

### Calibration and validation

We used field mosquito collection data during two periods, 1991–1996 and 2002–2003 [21], [33], in an area surrounding Barkedji village to *1)* calibrate and *2)* assess the goodness of fit of the population dynamics models using the coefficient of determination to measure how well the predicted *Ae. vexans* and *Cx. poicilipes* abundance values fit with a set of observed mosquito data. The latter were collected at Furdu and Niaka ponds near Barkedji village, every 20 days during the 2002 and 2003 rainy seasons (Figure 1b, Table 2) [34]. The mean number of *Culex* and *Aedes* collected per trap over the consecutive nights of a trapping session (between 5 and 9 days) was calculated. The mosquito population model was calibrated for the two species using 2002–2003 Furdu entomological data collection. The parameters identified as most sensitive by the sensitivity analysis were calibrated. The calibration was then performed with a systematic exploration of the input parameters space (Table 3). Other parameter values were determined based on literature data and expert knowledge (Table 1). To validate the model, we then compared observed and simulated relative abundances of *Ae. vexans* and *Cx. poicilipes* mosquito populations for the Niaka pond, 2002–2003 period. The degree of association between the temporal series was assessed by the calculation of the cross-correlation coefficient. This statistical index allows to test whether two temporal series are correlated. It returns values ranging from −1 (negative correlation) to 1 (positive correlation).

**Table 2:** Mosquito collections used for model calibration and validation, Barkedji, Senegal.

Year	Trap	No. trap-nights	Total *Aedes vexans*	Total *Culex poicilipes*	Reference
1991	C0_2_	37	6688	2780	[21]
1992	C0_2_	70	2654	1026	[21]
1993	C0_2_	79	1574	21213	[21]
1994	C0_2_	122	4756	4001	[21]
1995	C0_2_	80	12545	4964	[21]
1996	C0_2_	38	8114	2926	[21]
2002	Human baited	100	799	56	[33]
2003	Human baited	64	1106	468	[33]

**Table 3:** Calibration experimentation plan and resulting values.

Species	Parameter	Min	Max	Step	Result	No
***Culex poicilipes***	γ	0.81	0.99	0.02	0.99	10
	T	9	17	1	13	9
	Emax	7 10^5^	1.5 10^6^	10^5^	7 10^5^	9
	λ	100	200	20	150	3
***Aedes vexans***	γ	0.72	0.88	0.02	0.72	9
	T	3	10	1	7	8
	Td	5	7	1	7	3
	φ	0.83	0.99	0.02	0.98	9
***Total number of simulations***						6804

Between 1991 and 1996, mosquitoes were collected each year monthly between July and November in the Barkedji area with different kinds of traps at different locations [21] (Table 2). We computed the mean number of *Cx. poicilipes* and *Ae. vexans* collected per CO_2_ light trap and per night over the different locations. We used only one type of trap to avoid any trap related bias in the measure of mosquito abundance. CO_2_ light traps collections were used because those traps were used evenly each year. The degree of association between observed and simulated abundances for each mosquito species was assessed by calculating the cross-correlation coefficient.

### Simulation of *Aedes vexans* and *Culex poicilipes* populations from 1961 to 2003

Once validated, the models were run over a 63-year period, from 1961 to 2003, using rainfall historical records provided by the meteorological station of Linguère. As output, we considered the dynamics of each mosquito species expressed in relative values, as well as the product of the two temporal series. The latter index expresses the synchronicity of the *Ae. vexans* and *Cx. poicilipes* populations and higher values are obtained when the two mosquito populations are both abundant at the same time. It is subsequently referred as the Index of Simultaneous Abundance (ISA).

Finally, we compared and discussed the outputs of the model with the occurrence dates of RVF outbreaks or seroconversion rates reported in Northern Senegal and Southern Mauritania between 1987 and 2003 (Figure 1a) and with the annual prevalence rates recorded between 1989 and 2003 by the FAO sentinel herd system [50].

## Results

### Sensitivity analysis

**Figure 2:**
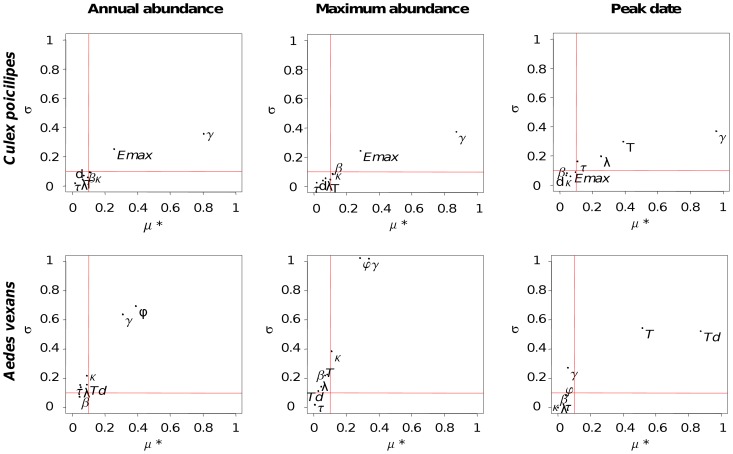
Sensitivity analysis results of the mosquito model. The graph represents the average of elementary effects in absolute values (μ*) according to their standard deviation (σ) to model outputs (cumulated annual abundance, maximum abundance, and date of the peak in abundance of *Culex poicilipes* and *Aedes vexans* mosquito populations). The red lines delimit the space in three types of parameters: i) those with negligible effects (μ*<0.1), ii) those with linear effects on the output, and without interaction between parameters (σ<0.1), iii) those with interactions and/or nonlinear relationship (μ*>0.1 and σ>0.1).

The sensitivity analysis (SA) allows identifying the key parameters of the population dynamics models for *Ae. vexans* and *Cx. poicilipes* species (Figure 2). Overall, the SA showed that the development period *T* and daily larval survival rate γ, which are both linked to the larval stage, are the parameters with the most effects on model outputs for the two species. Other parameters identified as influential for *Cx. poicilipes* were *Emax* and λ, two parameters concerning the oviposition, whereas the other key parameters for *Ae. vexans*, φ and *Td*, were related to the desiccation phase. These eight parameters were thus more accurately estimated through the calibration process.

### Calibration and validation

**Figure 3:**
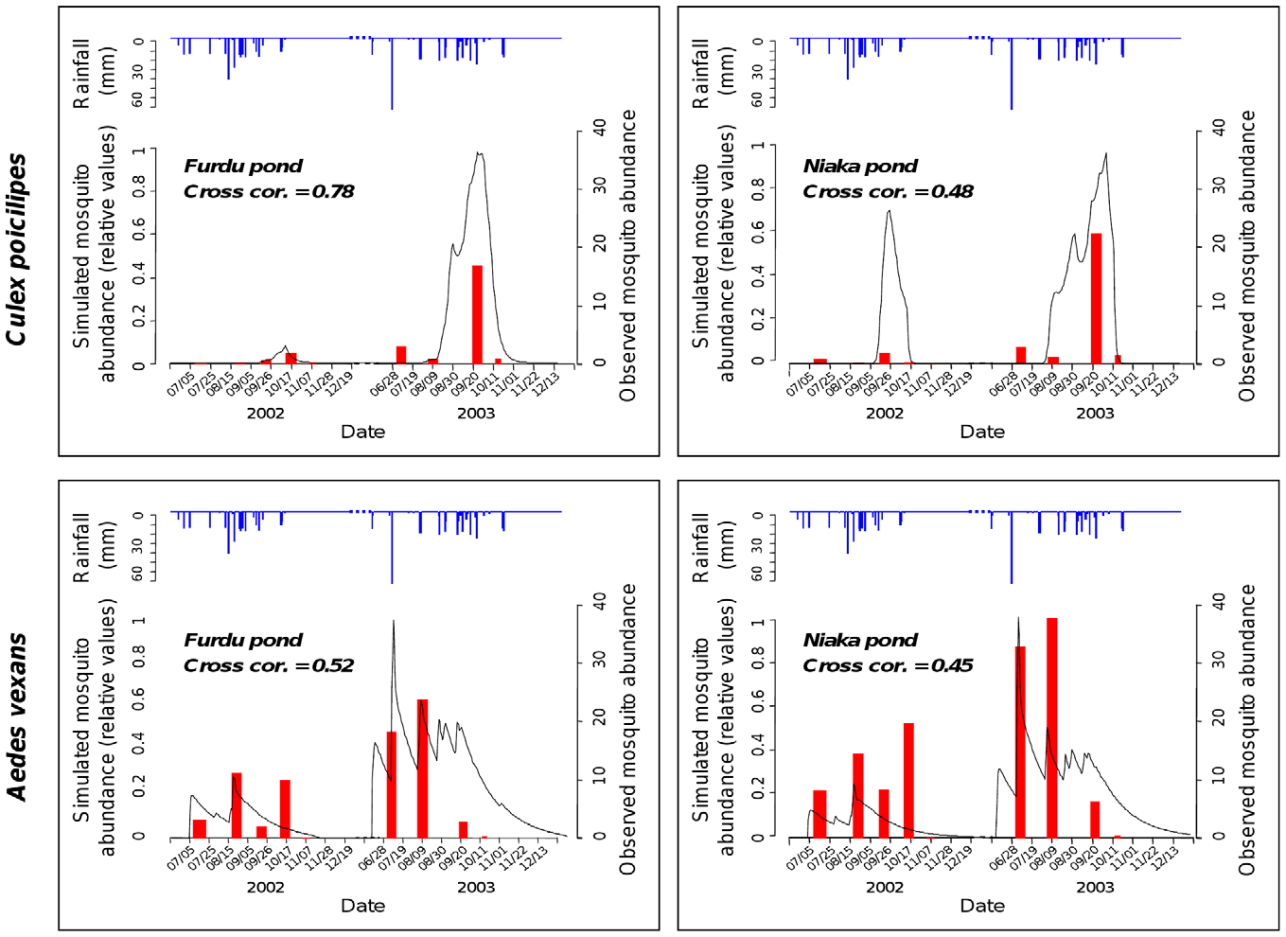
Simulated and observed mosquito abundances, Barkedji, Senegal, rainy seasons 2002 and 2003. *Culex poicilipes* and *Aedes vexans* observed mosquito abundance data are represented in red, simulated mosquito abundances are represented in black, and rainfall in blue.

The *T*, γ, *Emax*, λ, φ and *Td* parameter values were estimated from model calibration for *Cx. poicilipes* and *Ae. vexans* species on the Furdu pond (Table 2). The comparison of *Cx. poicilipes* and *Ae. vexans* observed abundances in 2002–2003 with outputs of the model showed that the model, driven only by rainfall data, reproduces well the major trends in the intra- and inter-annual population fluctuations (Figure 3). With cross-correlation values of 0.78 for *Culex*, to 0.52 for *Aedes*, the results of the simulations regarding the dates of the peaks and the proportion of abundance are consistent with entomological field data. When considering *Ae. vexans* populations, for both years the model reproduces well the first abundance peak of catches occurring at the beginning of the rainy season (July), generally after the first effective rainfall [33]. Moreover, the model simulates well the dates of maximum abundance at the end of the rainy season for *Cx. poicilipes* in 2002 and 2003. Finally, the model is able to correctly simulate the relative levels of abundance between the two years for the two species (higher *Cx. poicilipes* and *Ae. vexans* densities in 2003 than in 2002) (Figure 3).

**Figure 4:**
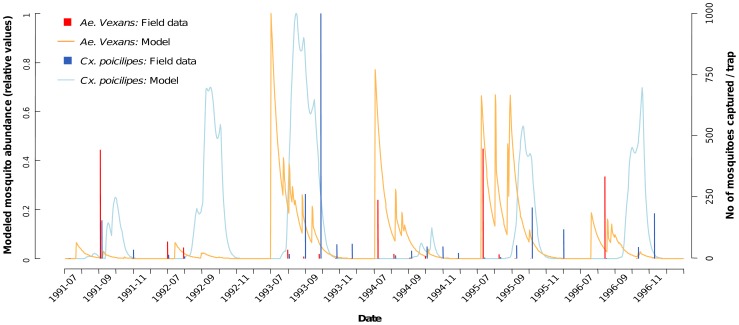
Simulated and observed *Culex poicilipes* and *Aedes vexans* mosquito abundance, Barkedji, Senegal, rainy seasons 1991–1996.

The comparison of observed and simulated mosquito abundances from 1991 to 1996 confirmed the capacity of the model to assess the inter-annual variability of *Cx. poicilipes* populations (Figure 4). For instance, the year of highest abundance of *Cx. poicilipes* observed during this six years period (1993) was clearly identified by the model. However, it failed to simulate the high abundances of *Ae. vexans* populations observed in 1991 and 1996 (Figure 4), suggesting that the model would only detect very high inter-annual variations in *Ae. vexans* abundances, like between the years 2002 and 2003. The cross-correlation coefficient values were fair (cor = 0.43 for both species). Finally, considering both population dynamics, the model reflects well the temporal interval between *Ae. vexans* and *Cx. poicilipes* dynamics, the former appearing at the very first rain, while the latter is stronger at the end of the rainy season, taking over from the declining *Ae. vexans* population.

### Simulation of *Aedes vexans* and *Culex poicilipes* populations from 1961 to 2003

**Figure 5:**
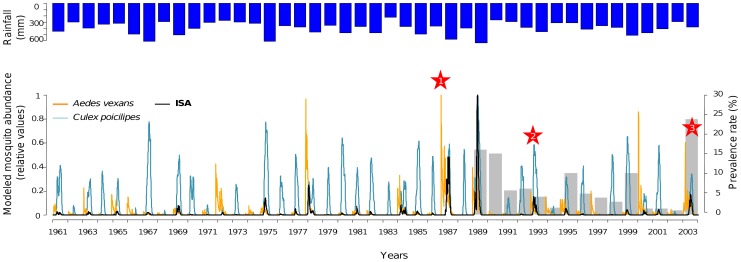
Modelled mosquito population dynamics, and Index of Simultaneous Abundance (ISA), Barkedji, Senegal, 1961–2003. Total rainfall per year is represented in blue. Modelled *Aedes vexans* population dynamics are represented in orange, modelled *Culex poicilipes* population dynamics are represented in dark blue. Gray bars indicate prevalence rate in sentinel herd as reported by the RVF surveillance system [56]. Stars indicate years with reported RVF outbreaks in Northern Senegal and Southern Mauritania: 1) In 1987, the RVF epizootic led to an epidemic among humans exposed to diseased animals, where more than 200 human deaths were recorded together with many abortions in livestock [25]; 2) In 1993, an increase of seroprevalence rates in livestock along the Senegal River was recorded [26]; 3) In 2003, five RVF outbreaks were reported in the Senegal River valley by the national RVF surveillance network, and high seroconversion rates were reported in small ruminants in Barkedji, Ferlo region [28], [57].

The modelled dynamics of *Ae. vexans* and *Cx. poicilipes* populations depict a high inter-annual variability over the studied period (Figure 5). Simulations put into evidence that the abundance of both species vary greatly between years. Moreover, the model shows that the peak of abundance of *Ae. vexans* populations generally occurs before the peak of *Cx. poicilipes* populations, depicting *Aedes*-before-*Culex* population cycles. Variations of the ISA reveal the variations in the temporal lag between *Ae. vexans* and *Cx poicilipes* populations.

The two major RVFV circulation events in northern Senegal and southern Mauritania were recorded in 1987 [25] and 2003 [28]. For these two years the model predicted high ISA values of *Ae. vexans* and *Cx. poicilipes* populations. According to this index, 1989 and 1993 also appear as years of simultaneous abundant mosquito populations (Figure 5). This is in agreement with the results of several sero-surveys conducted in the area. Serosurveys in small ruminants performed after 1988 showed an active transmission of RVFV till 1989 [26]. In October 1993, active RVFV transmission was detected in several locations of southern Mauritania, in association with an increase of abortions in small ruminant populations [26] (Figure 1a). That same year, RVFV was isolated from *Ae. vexans* and *Ae. ochraceus* species, and from one sheep in Barkedji village [27]. Between 1993 and 2003, no epizootic event was observed but virus circulation was detected in 1998 from *Cx. poicilipes* populations [29].

## Discussion

The results of our modelling approach are consistent with those of previous studies [21], [29], [34], [51], which argue that the two vector species *Ae. vexans* and *Cx. poicilipes* play a major synergistic role in RVFV transmission in Senegal, and that the years of high virus circulation levels coincide with years of high abundances of both mosquito species. In Figure 5 it can be seen that since 1961, years of RVF outbreaks do not coincide with years of highest total rainfall. Previous studies have shown that in West Africa, *Ae. vexans* and *Cx. poicilipes* abundance and total rainfall were not correlated [30], [31]. Rainfall variability was suggested to be more important than total rainfall for explaining mosquito populations, as the amount of *Ae. vexans* and *Cx. poicilipes* generation depends on the alternation of rainy and dry periods [33]. Our results come in support of these findings and suggestions, by providing evidence that present knowledge on the hydrology of temporary ponds and on mosquito population dynamics, as formalised in a model, is able to explain a large part of the observed mosquito abundance temporal variability. According to the yearly simulations, exceptionally high *Aedes* population densities were present in 1987 and 2003 (Figure 5). This result strengthens the hypotheses that RVFV may either be introduced by transhumant herds at the beginning of the rainy season or transmitted vertically in *Aedes* populations (which would explain the maintenance of the virus during inter-epizootic periods [21], [27], [28]), and would be amplified by *Aedes* populations, relayed by the *Cx. poicilipes* species [33], when both species are present abundantly at the same time. To a lesser extent, the same pattern can be observed in 1993 (Figure 5).

Due to the limited number of animals monitored, the RVF surveillance system showed limited capacities to correctly detect RVFV circulation and may have failed to detect animal cases [18], [28]. In 1993, RVF outbreaks were reported in Mauritania [26], whereas according to the surveillance system based on sentinel herds, only one sheep specimen was found infected in Barkedji in Senegal [27]. As confirmed by observation data [21], the small simulated *Ae. vexans* population may explain why no clinical cases were reported that year in Barkedji, suggesting again that the *Ae. vexans* population does play a major role in the amplification of the virus.

In 1987, the modelled mosquito abundances were the highest for the 1961–2003 period. In 1989, the *Ae. vexans* and *Cx. poicilipes* ISA was also very high, although no outbreak was detected. This can be explained by the probably high immunity rate of the ruminant populations following the 1987 outbreak, when animals may have been infected but remained asymptomatic cases. Moreover, since 1987 no other epizootic event led to an epidemic. Thus, although the simulated inter-annual variations in mosquito populations may explain the dates of RVF outbreaks observed between 1961 and 2003, others factors may drive the transition from an epizootic to an epidemic event. One strong possibility is the date of the Eid al-Kabir celebration, which favour very high ruminant concentrations [52], [53] and numerous contacts between humans and potentially viremic animals. Moreover, the co-occurrence in time of the *Ae. vexans* populations and the arrival of transhumant herds in the study area at the beginning of the rainy season may be crucial for the amplification of RVFV: if there are only few domestic ruminants available at the emergence of *Ae. vexans* populations, the virus will not spread.

Given the huge and dramatic socio-economic impacts of RVF, as well as its increasing global importance, there is an urgent need to develop appropriate mathematical tools for disease forecasting [18]. Our modelling approach which integrates presently available knowledge on RVF vector biology, is a first step towards the development of a climate-based early-warning system in Senegal which could allow prediction of at-risk periods for RVF, but certainly not the epidemic extent which is driven by human factors [54], [55].

Our results highlight that rainfall, as main driver of the hydrologic dynamics of the main breeding sites of RVF vectors, is a predictive factor of RVF in the studied area. In this respect, RVF in East and West Africa present very similar transmission processes, with water availability driving mosquito populations of the *Aedes* and *Culex* genera which have almost the same breeding sites and trophic behaviour [21].

More improvement on the model itself can be sought, as different simplifications have been made to develop a simple and robust model in a context of data poor areas. Improvements of the hydrological model have been discussed in [40]. To model the mosquito population dynamics, we considered water availability as the main constraint driving the population dynamics. Nevertheless, other variables, such as temperature, humidity, and vegetation cover, could be taken into account in the mosquito population model. These variables might impact the survival rates of mosquitoes in aquatic and aerial stages, as well as the RVFV development. Moreover, values of the different parameters, such as the date of diapause, could be better estimated from entomological data relative to *Ae. vexans* and *Cx. poicilipes* in Senegal.

### Concluding remarks

For the first time, mechanistic insight is provided in this study to explain why reported RVF outbreaks in Northern Senegal cannot be correlated directly to rainfall, as it is the case in East Africa. This is done through the use of a rainfall-driven model of RVF vector populations that combines a hydrological model to simulate daily water variations of mosquito breeding sites, with mosquito population models capable of reproducing the major trends of population dynamics of the two main vectors of RVFV in Senegal, *Ae. vexans* and *Cx. poicilipes*. Results show that RVF occurs during years when both species are present simultaneously in high densities. These occur when the rainfall temporal patterns result in water variations in the pond that are favourable for the reproduction of both mosquito species, *i.e.*, abundant rains occurring at regular intervals throughout the rainy season. The combined model can now be used in simulation studies for identifying which rainfall patterns would result in the simultaneous abundance of both species (high ISA), so that operational real-time rainfall-based monitoring systems can be developed.
